# Quo Vadis MRI?

**DOI:** 10.1016/j.zemedi.2024.12.002

**Published:** 2025-01-11

**Authors:** Jürgen Hennig

**Affiliations:** Freiburg, Germany

*Citius, altius, fortius* – faster, higher, stronger – the Olympic motto has been also true for the technological development of MRI over several decades. The first home-built whole-body systems by the early pioneers started with a field strength around 0.05 T – John Mallard’s Aberdeen magnet had 0.04 T [1], [2], Raymond Damadian’s huge superconductive magnet operated at 0.0503 T [3]. The first commercial systems already went to 0.35 T and quickly reached 0.5 T based on the conclusion in Paul Bottomley’s paper according to which RF-dephasing effects in the tissue would affect image quality beyond that field strength [4]. Still, already in 1983 the first 1.5 T system by GE came on the market and other manufacturers quickly followed suit.

During the ‘field strength war’ in the late 1980s, 0.5T and 1.5T systems coexisted, but eventually 1.5 T took over, although there were several studies that showed no statistically significant improvement in diagnostic outcome for the higher field [5], [6], [7], [8], [9], [10].

Low-field MRI based on permanent magnets with 0.2–0.4 T became limited to a niche market, mostly for orthopedic applications. Around 2003, 3 T systems became clinical, and the expectation was that – as before in the 0.5 vs. 1.5 T competition – 3 T would eventually take over, but this has not really happened yet; even today, 1.5 T is the dominant field strength in clinical applications. The original decision for going to 1.5 T was based on magnet construction considerations, as this was a field strength for which a magnet could be built with reliable quality and at a reasonable cost, but it has turned out to be a serendipitous choice: 1.5 T delivers good signal-to-noise and still is low enough to avoid RF issues at higher fields regarding SAR and B_1_ inhomogeneity.

In 2016, 7 T received FDA approval and is now available for patient applications. In the research arena, high fields naturally came sooner, Pierre Robitaille’s 8 T was already installed back in 1998 [11], around 2017 Kamil Ugurbil at CMRR got a 10.5 T system up and running [12], the 11.7 T ‘ISEULT’-system at Neurospin has just received approval for human scanning [13], [14], and recently the Dutch ultra-high field project to build a 14 T magnet was approved. In view of this continuous quest for higher and higher fields, it came as a surprise when Adrienne Campbell-Washburn published a paper in Radiology in 2019 on ‘Opportunities in Interventional and Diagnostic Imaging by Using High-Performance Low-Field-Strength MRI’, which showed very promising images acquired on a former 1.5 T system that had been down-ramped to 0.55 T [15]. Already in this first publication, she could demonstrate that with the improvements in all relevant components of the MRI hardware over the years, low-field MRI has now become a very valid supplement to the existing high-field systems. Improvements of the RF-chain have led to considerable better image quality, even without additional embellishment: 0.5 T images of today are nearly comparable to 1.5 T images from the early 1990s. Stronger and especially faster gradients allow to optimize data acquisition by reducing the time for the phase-encoding gradient and thus improve the sampling efficiency [16]. Most importantly, SNR has somewhat lost its terror by the introduction of AI-based noise filters. The combination of these technological advances has enabled low-field MRI (LF-MRI) to become an attractive supplement to conventional MRI, achieving a comparable diagnostic quality and patient throughput at least for those indications, which do not require very high spatial resolution. At low frequencies ≤ 30 MHz, the body is more or less transparent to RF, so that RF-heavy sequences like magnetic transfer (MT) can be freely used. Low Larmor frequency also means less susceptibility effects, which benefits imaging around metal implants. Most importantly, many implanted and interventional devices (pacemakers, perfusors, catheters, guidewires, biopsy needles, etc.) are MR-safe at this field and frequency without the need for costly modifications.

Currently, there are two commercial systems available at around 0.5 T. Synaptive Medical has developed a 0.5 T head-only MRI system with high-performance gradients for neuro applications [17]. The Siemens 0.55 T system has been designed for general use. Like the Synaptive system, it uses a helium-free magnet that allows easy installation with no helium re-fill and without the need for a quench pipe. The system’s comparably low cost, small size, low weight and small footprint makes it an attractive choice also in areas, where a ‘big’ conventional MRI system is not economically viable and thus – at least potentially – opens up new markets for MRI.

At the very low end, ultra-low field MRI (ULF-MRI), at a field strength still one order of magnitude lower, has also found new interest. The field strength of the home-built systems of the early pioneers has been around 0.05 T, and this regime is a current hot topic of research. Several groups have developed such systems, the first proof-of-principle magnet by Matt Rosen used an electromagnet [18], later ones were based on permanent magnets in either conventional pole shoe [19] or Halbach design [20]. It is clear that the very low intrinsic SNR at such field strength necessitates a radically different approach to MRI compared to its conventional clinical use. Data acquisition strategies have to be aimed to achieve the best possible SNR per unit time, which is mainly achieved by 3D acquisition. The rather short T_1_ at such low fields of around 400 ms for brain tissue allows shorter TR compared to higher fields, while the long T_2_ allows long echo trains, making 3D-RARE (FSE, TSE …) one of the preferred acquisition modes. Spin-echo based 3D acquisition also helps in the correction of the quite large field inhomogeneities inherent to simple magnet designs without high order shim coils. On the downside, concomitant gradients become significant even with modest gradient performance. With the help of AI-based reconstruction algorithms surprisingly decent image quality can be achieved within reasonable examination times [21]. At the low resonance frequency around 2–3 MHz, active electromagnetic noise cancellation can be used to avoid the necessity of a Faraday cage, making such systems truly mobile and light-weight [22].

The promise to bring MRI to application scenarios far outside the traditional venues in the more affluent parts of affluent countries has had a large impact even beyond the MR community. Hyperfine Inc. has launched its ‘Swoop’-system with a considerable amount of investment funding exceeding 300 million USD, which has been used not only for technological development, but especially to raise public awareness. On the one hand, this has brought the benefit of strong public attention; on the other hand it practically excludes commercial competition, as it appears rather hopeless to compete against such amount of funding.

The application scenario for ULF-MRI is – at least at present – not so much to compete with or supplement conventional MRI. In terms of practical applicability, the main competitor is ultrasound imaging (US). For the cost of a ULF-MRI system of ∼200 k€, one can get a very decent and versatile US system, which covers a broad range of clinical applications. So it appears that brain – which is not accessible to US – will remain the predominant area of application for ULF-MRI.

There are also very interesting recent developments for clinical MRI at very high fields beyond 3 T. 7 T has become a very useful tool for fMRI in neuroscientific applications, especially when the high field is combined with high performance gradients and excellent multichannel RF coils [23], [24]. In terms of clinical applications, it is still in the process to find its place. The detection of cortical dysplasia in epilepsy patients, not or hardly visible at lower fields, has so far been the most promising clinical indication where the high contrast-to-noise translates into significant clinical benefit. More general clinical applications are challenged by the severe RF issues at 300 MHz, which translates into a wavelength of around 10 cm in tissue. This leads to strong B_1_ inhomogeneities as well as to the risk of high local SAR. There has been tremendous progress to achieve reliable and robust imaging performance for neuroimaging, but outside the brain, 7 T MRI is still ‘work-in-progress’.

When clinical 3 T started 20 years ago, it was rather straightforward to adapt measurement protocols from 1.5 T to the changed relaxation rates at the higher field. Such adaptation is less straightforward when going from 3 T to 7 T: T_1_ becomes appreciably longer, T_2_ becomes shorter. Combined with the SAR issues, this necessitates quite significant changes to the measurement protocols at the higher field. As a consequence, 7 T needs specialized teams to operate. A further and more practical hindrance for the introduction of 7 T in clinical routine is the fact that a current 7 T scanner is still rather huge and does most often not fit into an existing examination room. Therefore, the 7 T is housed most often outside the radiological department in a separate building and operated by a separate team, which slows down the clinical adaptation. It has therefore been for several years my ‘ceterum censeo’ in numerous presentations on high-field MRI that high-field MRI will only have a chance for widespread clinical application if the system can be placed into an existing examination room with dimensions similar to the early 3 T scanners. 7 T will take a while to achieve this, but on several occasions, I hypothesized that a 5 T system could be a feasible goal. Meanwhile, a 5 T system has been realized (Jupiter MRI, United Imaging, Shanghai) and is already in clinical use [25], [26]. The images show that even coronal abdominal images look good, so the lower SNR compared to 7 T might be more than outweighed by the much broader range of clinical applications.

Not so long ago, clinical MRI was happening nearly exclusively at 1.5 T and 3 T with some niche applications – mainly orthopedic – for low-field permanent magnet systems. This has changed dramatically over the last few years. ULF-MRI and LF-MRI has (re)-entered the scene, and at the top end 5 T is filling the gap between 3 T and 7 T. This heterogeneity of field strengths has led to some re-labeling of the nomenclature. In the 1990s, 0.5 T was called low-field and high-field was reserved for 1.5 T. Now 0.5 T is labelled as mid-field in order to discriminate from low- and ultralow-field scanners. In the upper field strength range, the term ‘high-field MRI’ is now used for anything from 1.5 T to 7 T, depending on the context, and ultrahigh-field has been used for 7 T, 9.4 T, 10.5 T, 11.7 T to 14 T. The Finnish language has ∼ 40 different words for snow. The MR community needs to work on its vocabulary to discriminate between the quite extensive range of field strengths in use today. The question for the future will be how many field strength we really need. 0.05 T, 0.5 T and 1.5 T each have a very distinct application scenario and are expected to stay; at the higher field range, it is less clear. Will it be 3 T, 5 T, 7 T, 10+ T? Maybe 5 T (clinical ultrahigh-field) and 10+ T (for neuroscience) will be enough? Time (and economics) will tell.
